# Functional characterization of a terpene synthase responsible for (*E*)-β-ocimene biosynthesis identified in *Pyrus betuleafolia* transcriptome after herbivory

**DOI:** 10.3389/fpls.2022.1077229

**Published:** 2022-11-21

**Authors:** Xinzheng Huang, Hang Zhang, Huali Li, Mengting Wang, Xinyue Guo, Enliang Liu, Xiaoqiang Han, Congai Zhen, Aili Li, Wangpeng Shi, Yongjun Zhang

**Affiliations:** ^1^ Department of Entomology, MOA Key Lab of Pest Monitoring and Green Management, College of Plant Protection, China Agricultural University, Beijing, China; ^2^ State Key Laboratory for Biology of Plant Diseases and Insect Pests, Institute of Plant Protection, Chinese Academy of Agricultural Sciences, Beijing, China; ^3^ General Station of Agricultural Technology Extension, Xinjiang Production and Construction Corps, Urumqi, China; ^4^ Key Laboratory of Oasis Agricultural Pest Management and Plant Protection Resources Utilization, Xinjiang Uygur Autonomous Region, College of Agriculture, Shihezi University, Shihezi, China; ^5^ Institute of Grain Crops, XinJiang Academy of Agricultural Sciences, Urumqi, China

**Keywords:** wild pear, *Spodoptera litura*, plant-insect interactions, (*E*)-β-ocimene biosynthesis, plant defense

## Abstract

(*E*)-β-ocimene, a ubiquitous monoterpene volatile in plants, is emitted from flowers to attract pollinators and/or from vegetative tissues as part of inducible defenses mediated by complex signaling networks when plants are attacked by insect herbivores. Wild pear species *Pyrus betuleafolia* used worldwide as rootstock generally displays valuable pest-resistant traits and is a promising genetic resource for pear breeding. In the current study, transcriptional changes in this wild pear species infested with a polyphagous herbivore *Spodoptera litura* and the underlying molecular mechanisms were fully investigated. A total of 3,118 differentially expressed genes (DEGs) were identified in damaged pear leaf samples. *Spodoptera litura* larvae infestation activated complex phytohormonal signaling networks in which jasmonic acid, ethylene, brassinosteroids, cytokinin, gibberellic acid and auxin pathways were induced, whereas salicylic acid and abscisic acid pathways were suppressed. All DEGs associated with growth-related photosynthesis were significantly downregulated, whereas most DEGs involved in defense-related early signaling events, transcription factors, green leaf volatiles and volatile terpenes were significantly upregulated. The *PbeOCS* (GWHGAAYT028729), a putative (*E*)-β-ocimene synthase gene, was newly identified in *P. betuleafolia* transcriptome. The upregulation of *PbeOCS* in *S. litura*-infested pear leaves supports a potential role for *PbeOCS* in herbivore-induced plant defenses. In enzyme-catalyzed reaction, recombinant PbeOCS utilized only geranyl pyrophosphate but not neryl diphosphate, farnesyl pyrophosphate or geranylgeranyl diphosphate as a substrate, producing (*E*)-β-ocimene as the major product and a trace amount of (*Z*)-β-ocimene. Moreover, as a catalytic product of PbeOCS, (*E*)-β-ocimene showed repellent effects on larvae of *S. litura* in dual-choice bioassays. What is more, (*E*)-β-ocimene increased mortalities of larvae in no-choice bioassays. These findings provide an overview of transcriptomic changes in wild pears in response to chewing herbivores and insights into (*E*)-β-ocimene biosynthesis in pear plants, which will help elucidate the molecular mechanisms underlying pear-insect interactions.

## Introduction

Plants, especially perennial fruit trees such as pear, are frequently subject to attack by a variety of insect pests. In response to insect herbivore attack, plants have evolved highly sophisticated strategies allowing them to perceive various herbivory-associated molecules from insects’ oral secretions (or regurgitant), oviposition fluids or larval frass and trigger complex defense systems (Mao et al., 2022; Zhang et al., 2022). After the recognition of herbivores, plant initiate a series of early signaling events such as *V*m variation, Ca^2+^ elevation, production of reactive oxygen species and activation of MAP kinases, as well as phytohormone-centered regulatory networks to broad transcriptional reprogramming, ultimately leads to the synthesis and accumulation of defensive proteins and defense-associated secondary metabolites (Erb and Reymond, 2019). Cross-talk between different phytohormone signaling pathways help plants to fine-tune their responses to herbivore-specific cues. It is well known that the jasmonic acid (JA) pathway as a core route in plant defenses generally acts antagonistically toward the salicylic acid (SA) pathway (Erb and Reymond, 2019).

(*E*)-β-ocimene, a secondary metabolite commonly found in plants, plays important physiological and ecological roles in attracting pollinators and natural enemies of pests, as well as protecting plants directly against herbivores and pathogens (Shimoda et al., 2012; Su et al., 2022). This compound belongs to monoterpene which are derived from the methyl-erythritol-phosphate (MEP) pathway mainly in plastids and produced by terpene synthases (TPSs) using geranyl diphosphate (GPP) or neryl diphosphate (NPP) as the substrate. Several TPSs including (*E*)-β-ocimene synthase have been functionally characterized in many plants except for wild pear *Pyrus betuleafolia* (Chen et al., 2011; Jiang et al., 2019; Ding et al., 2020). According to phylogenetic relationships, plant TPS family fall into seven subfamilies including TPS-a, TPS-b, TPS-c, TPS-d, TPS-e/f, TPS-g and TPS-h, two of which, angiosperm TPS-b and TPS-g, consists mainly of monoterpenes (Karunanithi and Zerbe, 2019).

In modern pear production, cultivated species are generally used as scions, while wild species such as *P. betuleafolia* was commonly used as rootstocks (Dong et al., 2020). Native to China, *P. betuleafolia* is widely cultivated in pear orchards across the world due to its vigorous growth, high-affinity for different cultivars of Asian pears and European pears, and resistance or tolerance to several abiotic as well as biotic environmental stresses. It has thus been gaining more scientific attention, and its recently released genomic sequence (Dong et al., 2020) is an excellent resource for mining functional genes.

Pear trees emit more than 40 different volatile organic compounds, mostly composed of terpenes, esters, aldehydes and alcohols (Chen et al., 2020). Various terpenes are present in pear fruits, flowers and vegetative tissues. For instance, α-farnesene is a major terpenoid released from ripe fruits (Chen et al., 2006). Pear trees at the flowering and fruiting stages emit 14 terpenes including (*E*)-β-ocimene, caryophyllene, pinene, limonene and α-farnesene, which play crucial roles in the seasonal host shift of the lepidopteran pest *Grapholita molesta* from peach to pear trees (Najar-Rodriguez et al., 2013). (*E*)-β-ocimene and linalool emitted by flowers of *Pyrus bretschneideri* and *P. communis* were crucial for attracting honey bees (Su et al., 2022). Emissions of 17 terpenes including (*E*)-β-ocimene and α-farnesene are increased in leaves of pear trees after *pear psylla* infestation (Scutareanu et al., 2003), which is attractive to conspecifics of this insect (Horton and Landolt, 2007).

Transcriptional changes of *P. betuleafolia* in response to abiotic stresses such as drought and potassium deficiency have been well investigated (Li et al., 2016; Yang et al., 2020b). However, little is known about transcriptional changes in wild pears infested by herbivores. It was reported that PcAFS1, a sesquiterpene synthase in *Pyrus communis*, could catalyze the formation of α-farnesene attracting natural enemies of herbivores (Gapper et al., 2006). Yet, the biosynthesis of monoterpenoid including (*E*)-β-ocimene in pears, especially in *P. betuleafolia* has not been elucidated. Here, transcriptional changes in *P. betuleafolia* infested by lepidopteran *Spodoptera litura*, a notorious agricultural and horticultural pest, were investigated to figure out the molecular mechanisms underlying defense responses of pear plants to chewing insects and identify candidate TPSs responsible for terpene biosynthesis. Based on RNA-seq, *PbeOCS*, the (*E*)-β-ocimene synthase gene, was newly identified. Recombinant PbeOCS was then heterologously expressed in *Escherichia coli*, and its catalytic products were characterized using *in vitro* enzymatic assays. The impacts of (*E*)-β-ocimene on choice behavior and growth of *S. litura* larvae were also assessed. Our findings provide valuable insights in understanding of the molecular mechanisms underlying defense responses of pear plants against chewing insects and shed new light on the (*E*)-β-ocimene biosynthesis in pear plants.

## Materials and methods

### Plants and insects

One-year-old seedlings of *P. betuleafolia* planted in plastic pots (height, 30 cm; diameter, 35 cm) were placed in a greenhouse compartment. A month later, pear seedlings were used for further experiments. Eggs of *S. litura* was kindly provided by the Xinxiang experimental station of the Institute of Plant Protection, Chinese Academy of Agricultural Sciences (Henan Province, 35.09°N, 113.48°E). Insects were reared in artificial incubators at (26 ± 1)°C and 60% ± 10% humidity with a photoperiod of L:D=16:8 h.

### Plant treatment

Ten *S. litura* larvae were placed on one pear seedling in a nylon mesh cage at 18:00 PM. After 36 h infestation, larvae were removed from each plant, and the infested leaves were collected for subsequent RNA extraction. Non-infested seedlings were maintained under the same conditions and collected as controls. Three biological replicates were performed for each treatment.

### RNA extraction and RNA sequencing

Total RNA was extracted from seedlings leaves using EASYspin Total RNA Extraction Kit (Aidlab Biotechnologies Co., Ltd, Beijing, China), and assessed for quality using an Agilent 2100 Bioanalyzer (Agilent Technologies, CA, USA). A total amount of 1  μg RNA per sample was used for cDNA library preparation, and then was sequenced using a PE150 strategy on the Illumina Hiseq 2500 platform as described previously (Duan et al., 2021; Zhang et al., 2022). The clean reads were mapped to the wild pear *P. betuleafolia* genome (Dong et al., 2020). Genes with false discovery rate < 0.05 and log_2_(Foldchange) > 1 were used as differentially expressed genes (DEGs), which were functionally enriched to the Gene Ontology (GO) database using Blast2GO and KEGG pathways.

### qPCR measurement

The qPCR analysis was conducted using SuperReal PreMix Plus (SYBR Green) reagent kit (TianGen, Beijing, China) as described previously (Huang et al., 2018). The *tubulin* (accession number: AB239681) was used as an endogenous control. All primers are listed in **Table S1**. The qPCR analysis was carried out using the same RNA samples used for the RNA-seq.

### Cloning of PbeOCS and heterologous expression

Total RNA was extracted from herbivory-treated pear leaves to synthesize cDNA as described above for qPCR measurement. From the transcriptomic data, 43 putative TPS genes were annotated (**Table S2**), and the *PbeOCS* (GWHGAAYT028729) was cloned using specific primers (**Table S1**). For *in vitro* expression of *PbeOCS*, the ORF sequence was inserted into the expression vector pET-30a (+), and transformed into the *E. coli* strain BL21 (DE3), which were cultured at 37°C to an OD_600_ of 0.6 in 500 mL LB containing 100 μg/mL kanamycin. Then, the cultures were induced by addition of 1 mM isopropyl 1-thio-β-d-galactopyranoside and incubated at 18°C and 150 rpm for 20 h. Subsequently, cells were harvested by centrifugation, resuspended in extraction buffer and disrupted by sonication (Zhang et al., 2022).

### *In vitro* enzymatic assay

Enzymatic activity assays of the recombinant PbeOCS were performed using geranyl pyrophosphate (GPP), neryl diphosphate (NPP), farnesyl pyrophosphate (FPP) and geranylgeranyl diphosphate (GGPP) as substrates (Huang et al., 2018; Zhang et al., 2022). Reaction mixtures consisted of 500 μL of *E. coli* crude extract, 10 μM substrate, 10 mM MOPS (pH 7.0), 10 mM MgCl_2_, 0.2 mM Na_2_WO_4_, 0.05 mM MnCl_2_ and 0.1 mM NaF, 1 mM dithiothreitol and 10% (v/v) glycerol. Samples were incubated at 30°C for 1 h, and the volatile products were trapped by solid phase microextraction (SPME) with a 65 μm polydimethylsiloxane (PDMS) fiber for 30 min at 30°C, and then analyzed by a GC-MS system (GCMS-QP2010 SE, Shimadzu, Kyoto, Japan) fitted with an Rxi-5Sil column (30 m × 0.25 mm × 0.25 μm) (Qi et al., 2018; Zhang et al., 2022). The PbeOCS product was identified by comparing retention time and mass spectrum with that of the authentic (*E*)-β-ocimene standard (CAS: 3779-61-1, J&K Chemical Company, Beijing, China) under the same conditions. Meanwhile, bacterial cells harboring empty vector pET-30a (+) were used as the negative control.

### Effect of (E)-β-ocimene on larval behavior and survival rates of *S. litura*

The (*E*)-β-ocimene was dissolved in hexane at 0.1 μg/μL, 1 μg/μL and 10 μg/μL. The behavioral choices of 3^rd^ instar *S. litura* larvae to (*E*)-β-ocimene were assessed in a 9 cm diameter plastic petri dishe as described by previous studies (Guo et al., 2021; Zhang et al., 2022). Each concentration was tested with 30 *S. litura* larvae, and five biological replicates were performed. After 2 hours, the choice of *S. litura* larvae was recorded.

Moreover, survival rate of larvae exposed to (*E*)-β-ocimene (0.1 μg/μL, 1 μg/μL and 10 μg/μL), dissolved in 20 μL hexane and added to the 500 mg of artificial diet, was also recorded. Artificial diet containing n-hexane was employed as the control. Newly hatched larvae were reared on the artificial diet in 24-well plates (one larva per well) and allowed to feed for 9 consecutive days. Each concentration was tested with 24 larvae, and three biological replicates were performed.

### Statistical analysis

All data were analyzed using SPSS Statistics software (version 17.0) (SPSS Inc., Chicago, IL, USA). Data are presented as the mean ± SEM. Gene expression data and survival rate data were analyzed using Student’s *t*-test. Larval choice data were subjected to a χ^2^ test (50:50 distribution) to determine any differences between controls and treatments.

## Results

### Transcriptome analysis of *P. betuleafolia* induced by *S. litura*

After assembly, 60,965 genes were found in infested and non-infested leaves of *P. betuleafolia*. Based on transcriptomic data, a principal component analysis plot was constructed (**Figure S1**). A total of 3,118 DEGs were identified in *P. betuleafolia* leaves induced by *S. litura* infestation. Among these DEGs,1,545 were significantly upregulated and 1,573 were significantly downregulated (**Figure S2**; **Table S3**).

**Figure 1:**
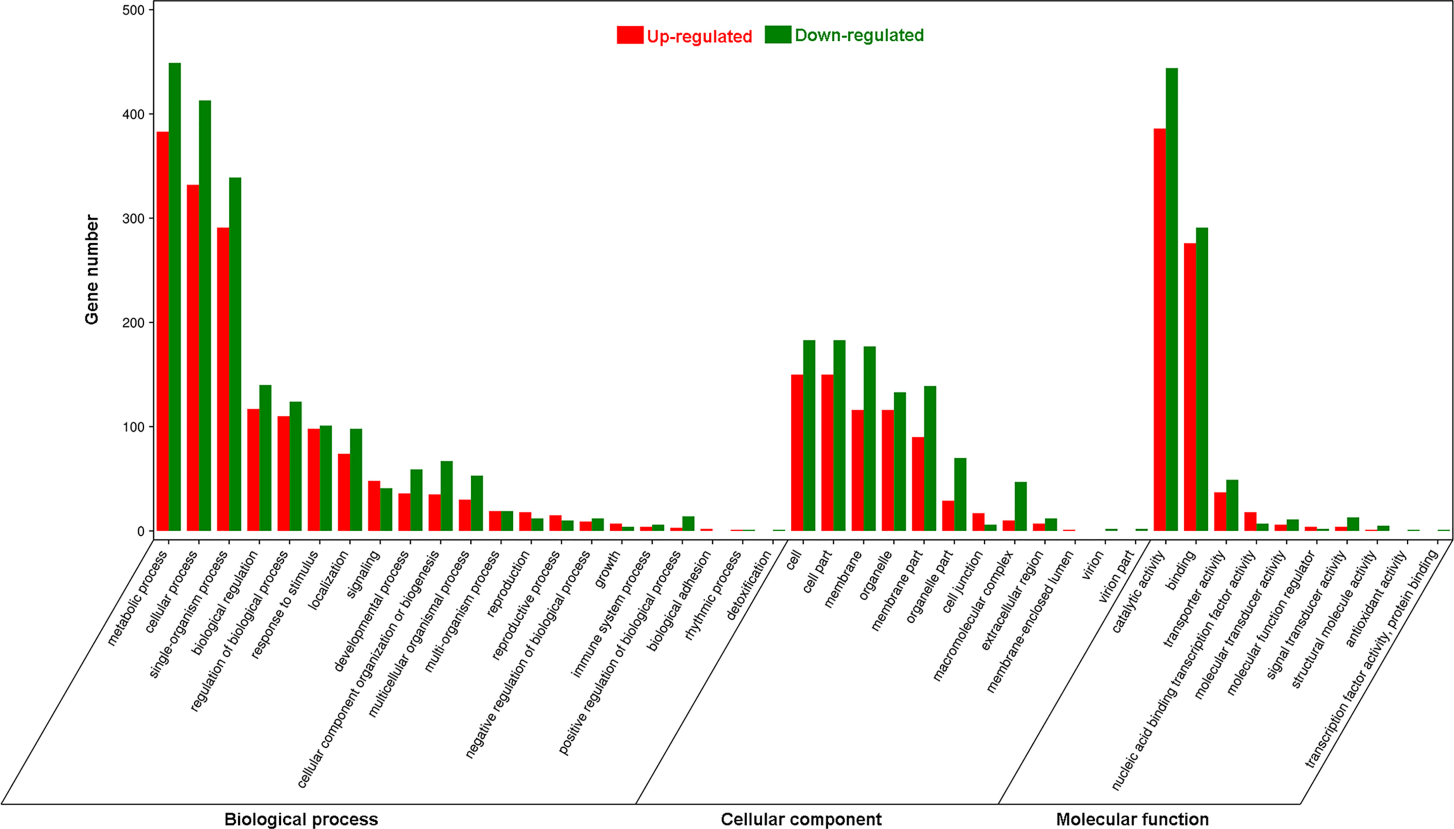
Gene Ontology (GO) analysis of differentially expressed genes (DEGs) in *Pyrus betuleafolia* leaves after *Spodoptera litura* infestation.

**Figure 2:**
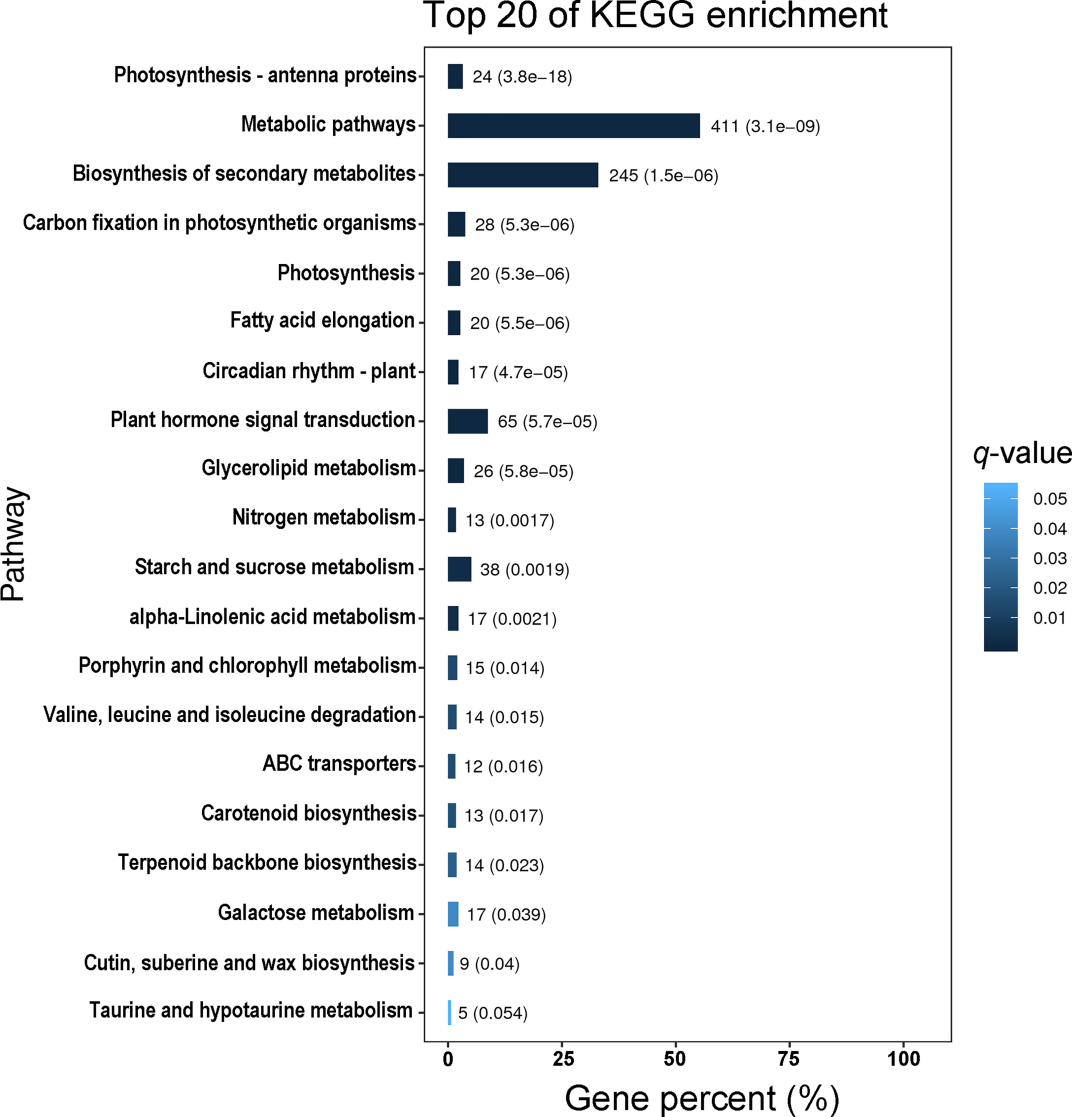
The top 20 KEGG pathways enriched with differentially expressed genes (DEGs) in *Pyrus betuleafolia* leaves after *Spodoptera litura* infestation.

Using Gene Ontology (GO) analysis, these detected DEGs were classified into three GO categories: biological process, cellular component and molecular function (**Figure 1**). The top three terms within biological process category were metabolic process (GO 0008152; 383 up- and 449 downregulated genes), cellular process (GO 0009987; 332 up- and 413 downregulated genes), and single-organism process (GO 0044699; 291 up- and 339 downregulated genes). The most abundant terms in category of cellular component were cell (GO 0005623; 150 up- and 183 downregulated genes), and cell part (GO 0044464; 150 up- and 183 downregulated genes), followed by membrane (GO 0016020; 116 up- and 177 downregulated genes), organelle (GO 0043226; 116 up- and 133 downregulated genes) and membrane part (GO 0044425; 90 up- and 139 downregulated genes). Most DEGs in molecular function ontology were involved in catalytic activity (GO 0003824; 386 up- and 444 downregulated) and binding (GO 0005488; 276 up- and 291 downregulated). In the KEGG pathway analysis to identify pathways activated by *S. litura* infestation, the top three KEGG pathways were metabolic pathways (411 genes), biosynthesis of secondary metabolites (245) and plant hormone signal transduction (65) (**Figure 2**).

**Figure 3:**
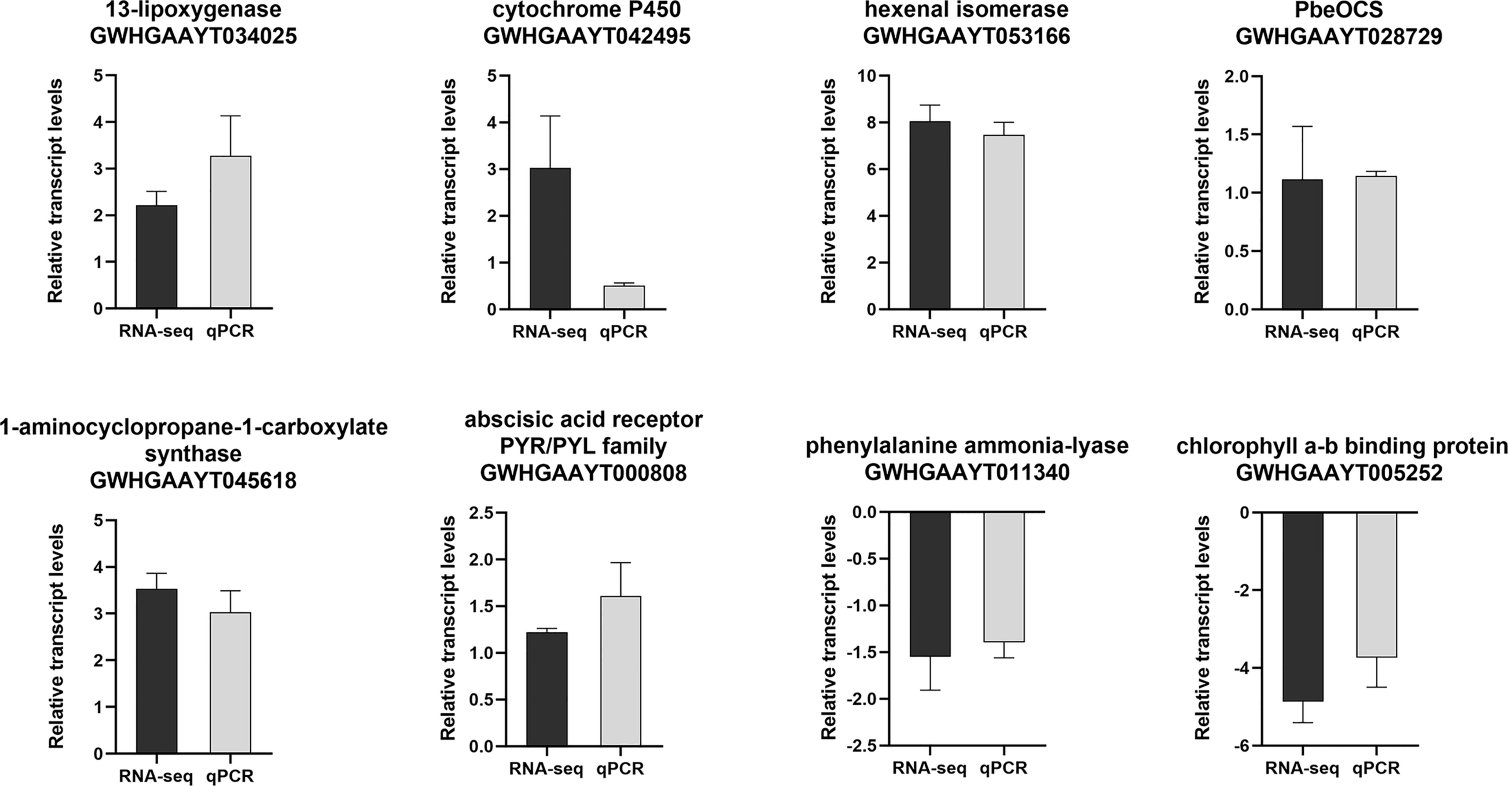
The relative expression levels of selected genes from the RNA-seq (black bar) and qPCR (gray bar) analysis.

Eight DEGs involved in JA pathway, SA pathway, ethylene pathway, abscisic acid pathway, photosynthesis, terpene volatiles biosynthesis and the green leaf volatiles biosynthesis were selected for qPCR validation. Based on these results, these DEGs were highly consistent with the data from the RNA-seq (**Figure 3**), indicating the reliability and repeatability of the RNA-seq data.

### DEGs involved in phytohormone pathways

Nineteen genes associated with JA biosynthesis pathway and 13 genes related to the JA signaling pathway were differentially expressed in *S. litura-*infested *P. betuleafoli*a leaves, and most (29 of 32) of these genes were significantly upregulated. In contrast, genes associated with SA (6 of 8) were mostly downregulated (**Table S4**). In addition, most of the DEGs related to ethylene (10 of 12), brassinosteroids (6 of 6), cytokinin (6 of 11), gibberellic acid (5 of 8) and auxin (23 of 41) were upregulated, whereas genes involved in abscisic acid (22 of 33) were mostly downregulated (**Table S4**).

### DEGs involved in early signaling events and transcription factors (TFs)

Several DEGs related to Ca^2+^ signaling (3 up- and 4 downregulated), reactive oxygen species (28 up- and 19 downregulated) and MAP kinases (7 up- and 3 downregulated) were present in leaves of *P. betuleafolia* after *S. litura* infestation (**Table S5**). There were 135 genes annotated as TFs were differentially expressed after *S. litura* infestation, amongst of which 93 genes were upregulated and 42 genes were downregulated. Most DEGs belonged to the gene family bHLH (16 up- and 13 downregulated), followed by AP2-EREBP (19 up- and 9 downregulated genes) and WRKY (21 up- and 2 downregulated) (**Table S6**).

### DEGs associated with primary and secondary metabolism

Many genes involved in amino acid metabolism (51 up- and 33 downregulated), carbohydrate metabolism (79 up- and 81 downregulated) and nucleotide metabolism (10 up- and 7 downregulated) were differentially expressed after *S. litura* infestation. Additionally, all of the 44 DEGs related to photosynthesis were significantly downregulated (**Table S7**).

Numerous genes related to cytochrome P450 (CYP, 30 up- and 34 downregulated) and UDP-dependent glucosyltransferases (UGTs; 7 up- and 11 downregulated) that contribute to pest and disease resistances were differentially expressed (**Table S8**). Moreover, many DEGs associated with volatile secondary metabolite biosynthesis were present in *P. betuleafolia* leaves after infestation. Although all six DEGs for phenylpropanoid or benzenoid biosynthesis were downregulated, eight of 11 DEGs involved in green leaf volatiles production were upregulated (**Table S8**), including two hexenal isomerase genes involved in the conversion of (*Z*)-3-hexenal to (*E*)-2-hexenal (Spyropoulou et al., 2017). Most DEGs related to terpenoid volatiles biosynthesis (13 of 18 DEGs), including six TPSs, were significantly upregulated (**Table S8**).

### Functional characterization of PbeOCS

**Figure 4:**
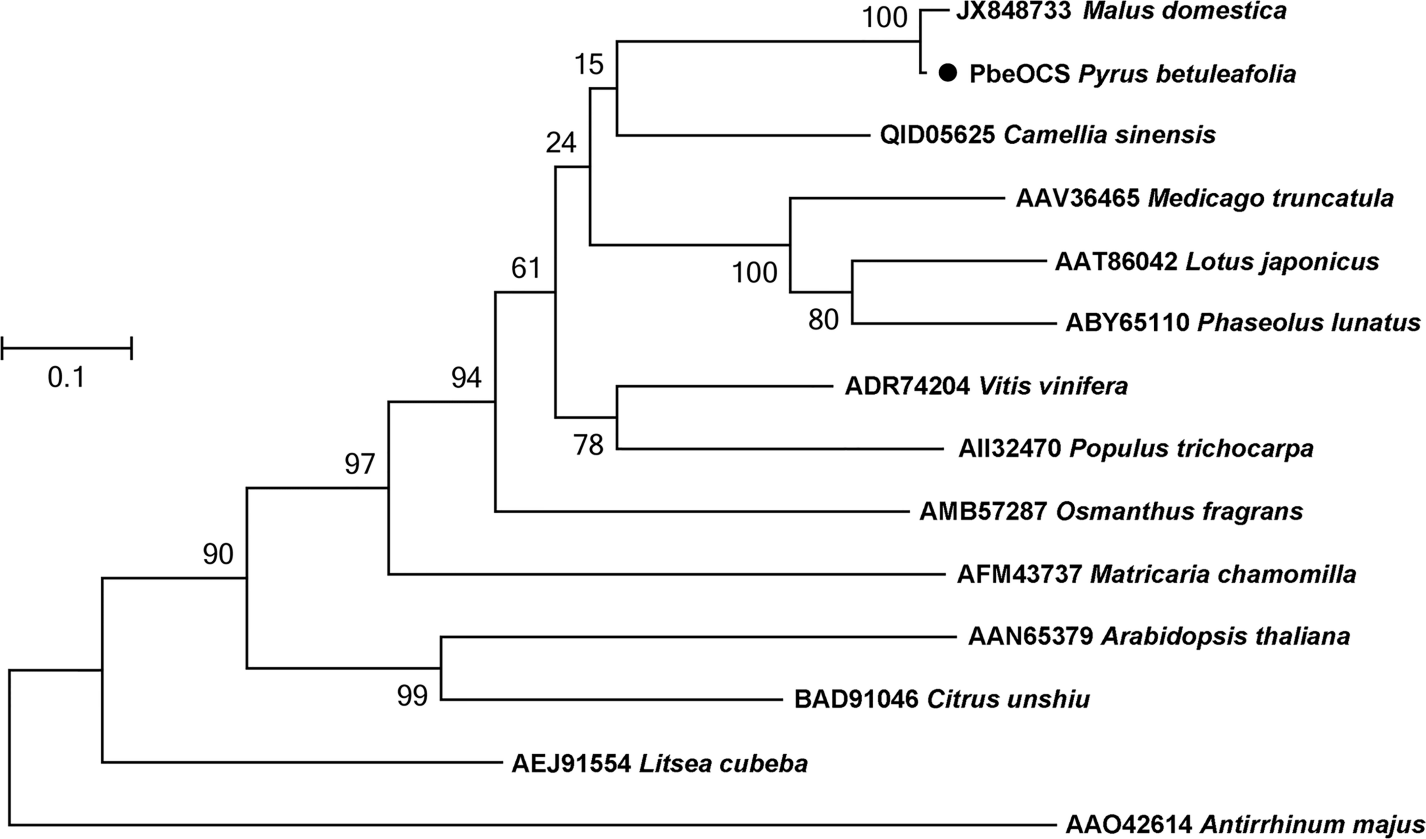
Phylogenetic tree of amino acid sequences of PbeOCS from *Pyrus betuleafolia* and *(E)*-β-ocimene synthase from other plants. The tree was generated using the maximum-likelihood method with 1,000 bootstrap replications.

A monoterpene synthase gene *PbeOCS* (GWHGAAYT028729) was identified in the transcriptome of *P. betuleafolia* after *S. litura* infestation. The *PbeOCS* had a 1647-bp ORF encoding 548 amino acids with a deduced molecular mass of ca. 62.65 kDa. Phylogenetic analysis showed that *PbeOCS* shared highest similarity (97.26% identity) with the (*E*)-β-ocimene synthase gene in *Malus domestica* (accession no: JX848733) (Nieuwenhuizen et al., 2013), followed by the (*E*)-β-ocimene synthase gene in *Camellia sinensis* (65.28%; accession no: QID05625) (**Figure 4**) (Chen et al., 2021). The PbeOCS protein has conserved structural features such as the DDxxD motif, the NSE/DTE motif, the RS(X)_8_W motif in the N-terminal region, and the arginine-rich RxR motif located at 35 amino acids upstream of the DDxxD motif, all of which are highly conserved in TPSs in plants (Karunanithi and Zerbe, 2019). ChloroP and TargetP showed that PbeOCS sequence does not have a signal peptide. The qPCR analysis revealed that *PbeOCS* was upregulated after *S. litura* infestation.

**Figure 5:**
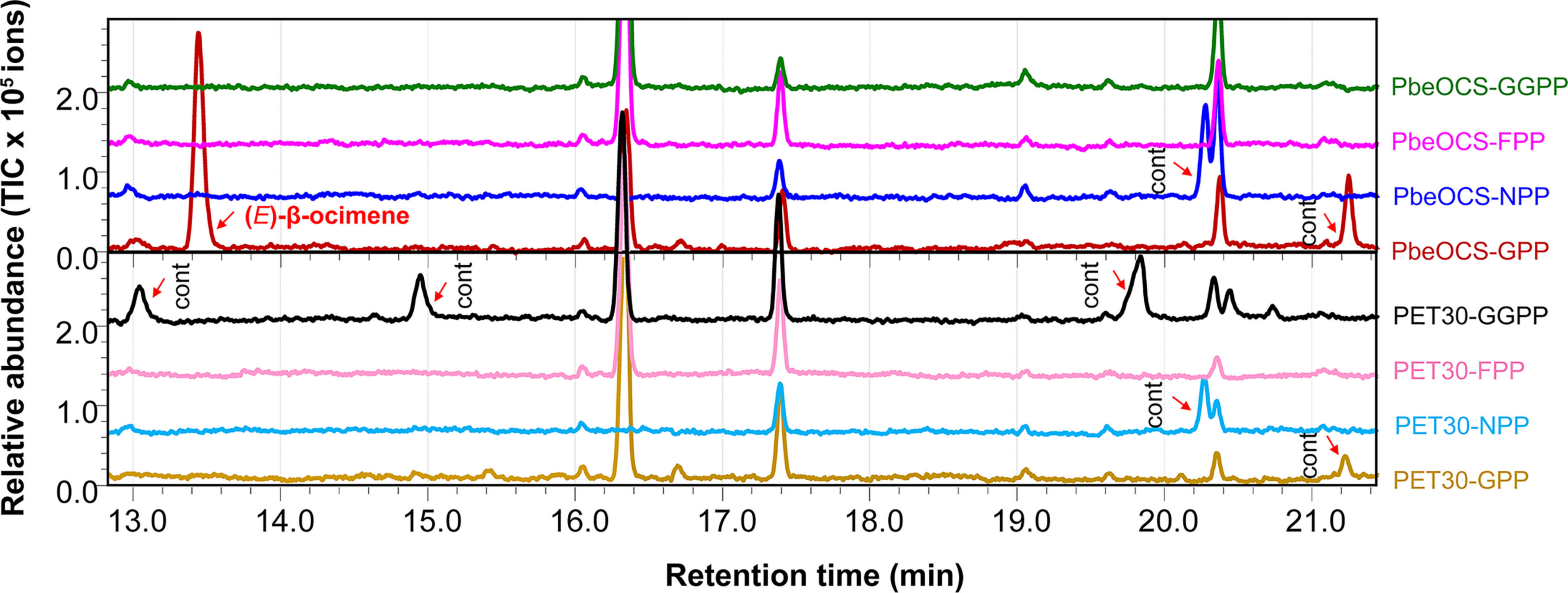
*In vitro* enzymatic assays of recombinant PbeOCS using different substrates. Cont, contamination.

The recombinant PbeOCS *in vitro* only used GPP as the substrate to produce (*E*)-β-ocimene as the major product (**Figure 5**) and a trace amount of (*Z*)-β-ocimene detected in the m/z 93 spectrum (not shown in the **Figure 5**). When NPP, FPP and GGPP were provided as substrates, no product was detected. Meanwhile, cells harboring empty vector did not yield any detectable terpene product (**Figure 5**).

### Larval preference for (E)-β-ocimene and herbivore performance test

**Figure 6:**
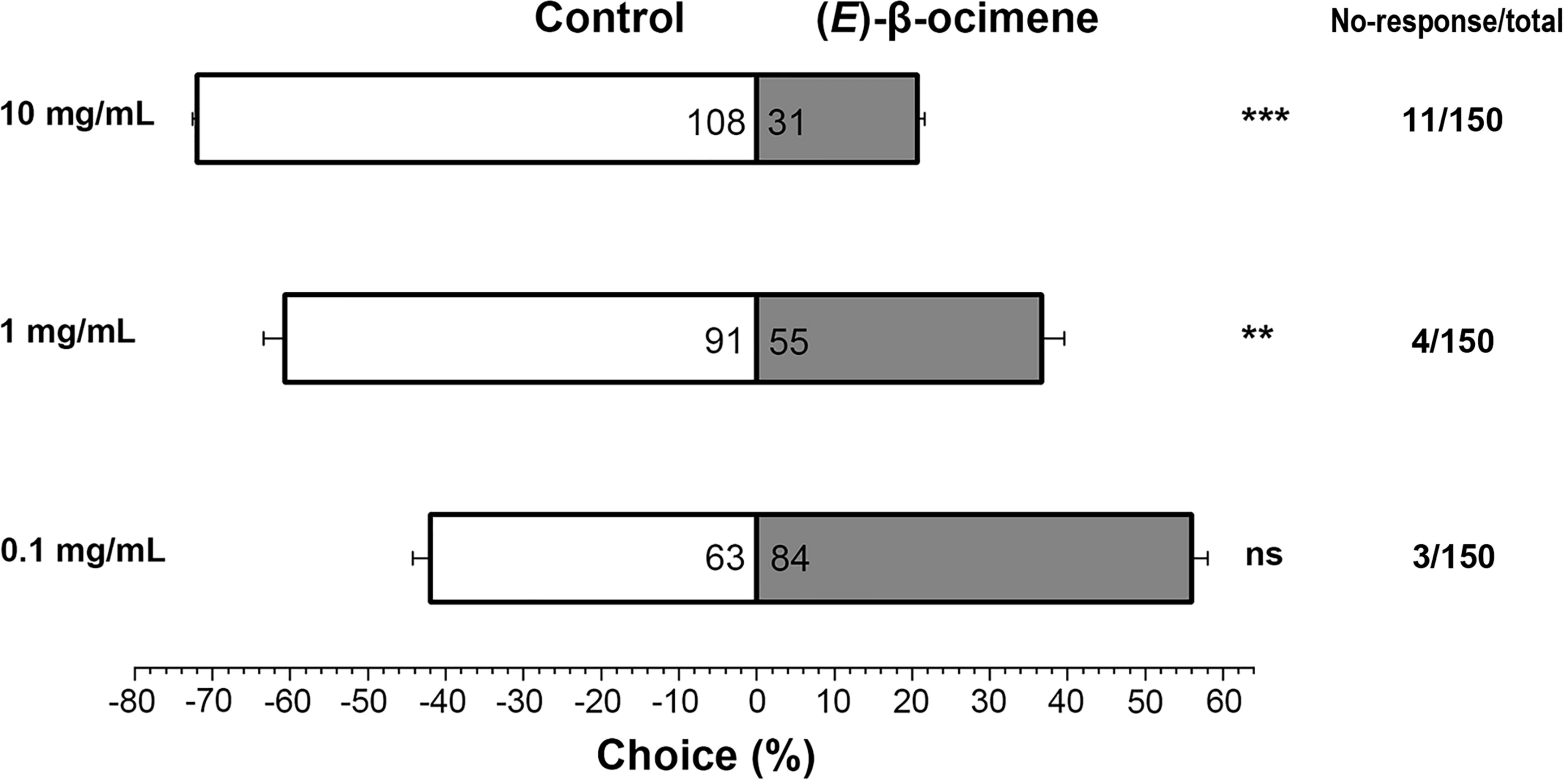
Behavioral preference of *Spodoptera litura* larvae for (*E*)-β-ocimene. Data were tested for significant differences using a χ^2^ test **P < 0.01; ***P < 0.001; ns, no significance.

**Figure 7:**
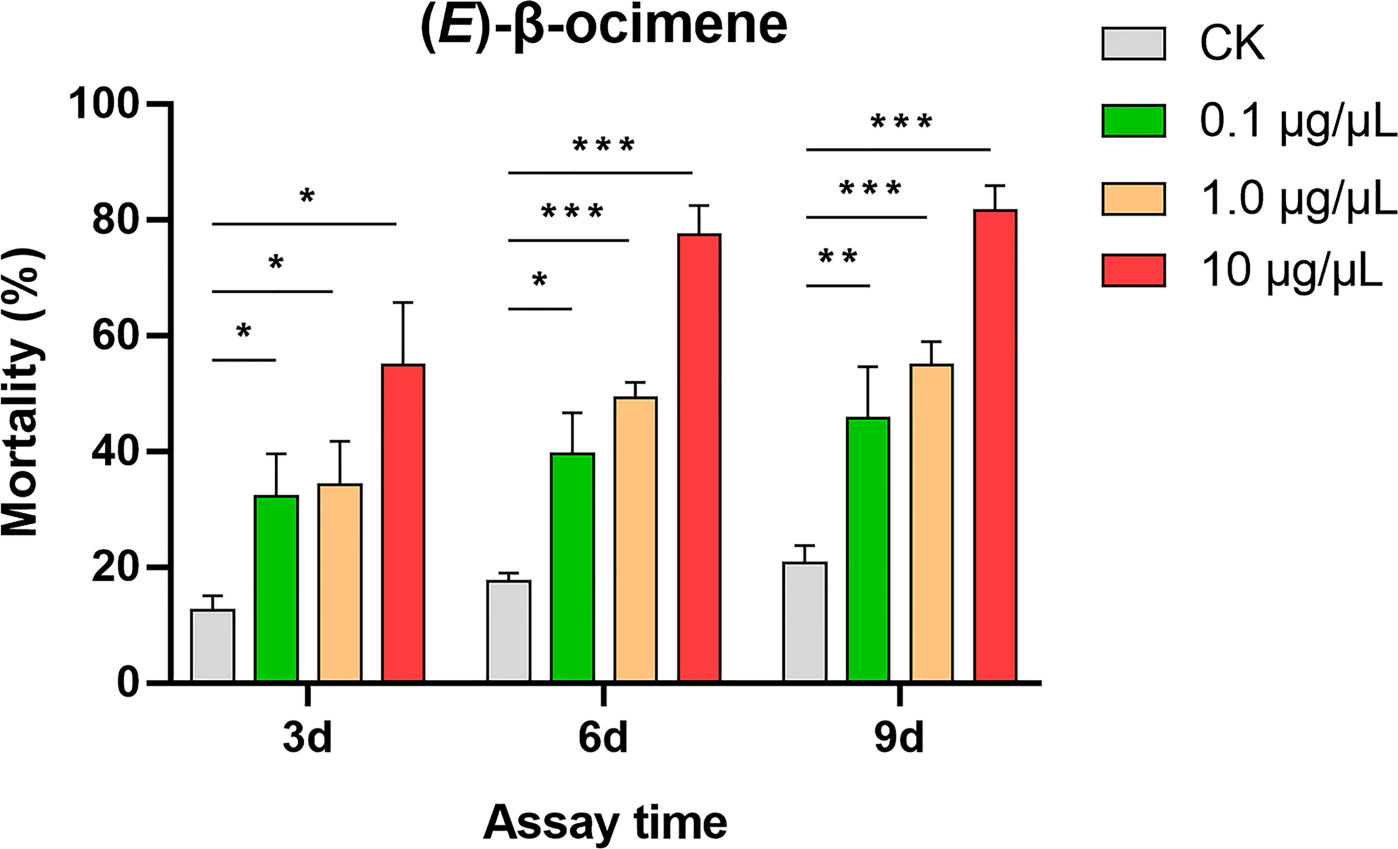
Larval mortality of *Spodoptera litura* caused by (*E*)-β-ocimene. Data were tested for differences using Student’s *t*-test (*P < 0.05; **P < 0.01; ***P < 0.001).

In the dual-choice bioassay, *S. litura* larvae showed significantly preference for control diet than those containing (*E*)-β-ocimene at 10 μg/μL (χ^2^ = 42.655, *P* < 0.001) or 1 μg/μL (χ^2^ = 8.877, *P* = 0.003), although larvae did not display a significant response to 0.1 μg/μL (*E*)-β-ocimene (χ^2^ = 3.000, *P* = 0.083; **Figure 6**). In the no-choice bioassay, the *S. litura* larvae feeding on the diet treated with different concentration of (*E*)-β-ocimene (0.1 μg/μL, 1 μg/μL and 10 μg/μL) displayed significantly higher mortalities compared to those feeding on control diet (0.1 μg/μL: 3d, *P* = 0.018; 6d, *P* = 0.011; 9d, *P* = 0.036; 1.0 μg/μL: 3d, *P* = 0.028; 6d, *P* < 0.001; 9d, *P* < 0.001; 10 μg/μL: 3d, *P* = 0.005; 6d, *P* < 0.001; 9d, *P* < 0.001; **Figure 7**).

## Discussion

In this study, transcriptional changes in *P. betuleafolia* in response to chewing *S. litura* were investigated. Phytohormone biosynthesis and signaling pathways have been reported to play a central role in mediating plant defense responses against herbivores attack through linking perception of herbivore-derived stimuli and early signaling networks to broad downstream transcriptional reprogramming and defense induction (Erb and Reymond, 2019). Different herbivores often trigger distinct transcriptional responses and signal-transduction pathways in plants. Generally, chewing herbivores such as caterpillars and cell-content feeders such as thrips and mites activate JA pathway, while phloem-feeding insects such as whiteflies and aphids trigger SA pathway (Stam et al., 2014). SA signaling and JA signaling pathways often act through negative crosstalk. So not surprisingly, our findings consistently showed that chewing *S. litura* larvae activated JA signaling pathway while SA-dependent defenses were suppressed (**Table S4**). Moreover, other phytohormone pathways such as ethylene, brassinosteroids, cytokinin, gibberellic acid, auxin and abscisic acid were also involved in pear defenses against *S. litura* (**Table S4**). These findings indicated that *S. litura* larvae infestation triggered complex phytohormone signaling networks in pear plants, and crosstalk between these phytohormones might allow the pear seedlings to fine-tune its induced defense response to specific herbivores.

TF families are known to play important roles in herbivore-induced direct and indirect defenses in plants, which regulate the transcript levels of the target genes and potentiate defense responses (Erb and Reymond, 2019). In the present study, 135 TFs, primarily bHLH (29 genes), AP2-EREBP (28 genes) and WRKY (23 genes), were differentially expressed in *S. litura*-infested *P. betuleafolia* leaves (**Table S6**). Several TFs involved in the transcriptional regulation of insect-induced volatile terpenes, including (*E*)-β-caryophyllene in *Arabidopsis thaliana* (Hong et al., 2012), (*E*)-β-farneseneas and (*E*)-α-bergamotene in *Zea mays* (Li et al., 2015) linalool in *Freesia hybrida* (Yang et al., 2020a), and *Dendrobium officinale* (Yu et al., 2021), valencene and (*E*)-geraniol in *Citrus sinensis* (Shen et al., 2016; Li et al., 2017) and α-farnesene in *M. domestica* (Wang et al., 2020), have been identified and functionally characterized. However, the role of TFs in terpene biosynthesis in pear is still not clear and need further investigation.

Many genes involved in biosynthesis of defensive metabolites, such as CYP (64 genes) and UGTs (18 genes), were differentially expressed (**Table S8**). In addition, 24 genes associated with the biosynthetic pathways of volatile terpenoids and HPL-derived green leaf volatiles were upregulated after *S. litura* infestation, highlighting their potential roles in *S. litura*-induced pear defenses. Unlike defense-related nonvolatile and volatile secondary metabolites, growth-related photosynthesis was suppressed in *S. litura*-infested *P. betuleafolia* (**Table S7**), which is similar to the findings reported in the defense response of potato (Mao et al., 2022), cotton (Zhang et al., 2022), rice (Wang et al., 2020; Satturu et al., 2021), wheat (Zhang et al., 2020) and tomato (Ke et al., 2021) plants induced by herbivores. In herbivore-damaged plants, suppression of photosynthesis might be a general phenomenon as a result of a trade-off between growth and defense, which allow plants to reallocate its limited resources toward activation of anti‐herbivore defense responses and production of inducible secondary metabolites (Zhou et al., 2015).

In plants, (*E*)-β-ocimene is one of the most common volatiles that act as an attractant in flower-pollinator interactions, a defensive compound in leaf-pest interactions and/or a priming signal in plant-plant communication (Fernández-Milmanda, 2022; Onosato et al., 2022). (*E*)-β-ocimene in flower and leaves of pear has multiple ecological roles in attracting pollinators and plant-pest interactions (Scutareanu et al., 2003; Su et al., 2022). The TPS in catalyzing (*E*)-β-ocimene biosynthesis, namely (*E*)-β-ocimene synthase, has been functionally characterized in several plant species such as *Antirrhinum majus* (Dudareva et al., 2003), *Arabidopsis thaliana* (Fäldt et al., 2003), *Camellia sinensis* (Chen et al., 2021; Jian et al., 2021), *Citrus unshiu* (Shimada et al., 2005), *Lotus japonicus* (Arimura et al., 2004), *Malus domestica* (Nieuwenhuizen et al., 2013), *Matricaria recutita* (Irmisch et al., 2012), *Medicago truncatula* (Navia-Giné et al., 2009), *Morus notabilis* (Ding et al., 2020), *Nicotiana attenuata* (Xu et al., 2020), *Osmanthus fragrans* (Zeng et al., 2015), *Phaseolus lunatus* (Arimura et al., 2008), *Populus trichocarpa* (Irmisch et al., 2014) and *Vitis vinifera* (Martin et al., 2010). In the current study, the (*E*)-β-ocimene synthase gene *PbeOCS* was present in the RNA-seq data and functionally characterized *in vitro* (**Figure 5**). The upregulation of *PbeOCS* after *S. litura* infestation suggested that *PbeOCS* and its enzymatic product (*E*)-β-ocimene are likely to be involved in insect-induced pear defenses. Interestingly, subsequent insect bioassay revealed that (*E*)-β-ocimene had a repellent effect on *S. litura* larvae (**Figure 6**) and significantly increased larval mortality (**Figure 7**). Additionally, candidate *TPSs* involved in the biosynthesis of α-pinene, limonene, linalool, germacrene D, β-caryophyllene (3*E*)-4,8-dimethyl-1,3,7-nonatriene (DMNT) and (*E*,*E*)-4,8,12-trimethyl-1,3,7,11-tridecatetraene (TMTT) were identified in our transcriptomic data, which still need to be further functionally characterized.

In this study, transcriptomic analyses of wild pear *P. betuleafolia* after *S. litura* infestation were performed to elucidate the molecular mechanisms underlying pear-herbivore interactions and to mine new TPSs. Our results revealed that *S. litura* infestation triggered a drastic and extensive transcriptomic reprogramming in pear leaves, including activation of a series of early signaling events and induction of complex phytohormone signaling networks. Significantly, almost all DEGs in the JA pathway were significantly upregulated, indicating that the chewing *S. litura* mainly activated JA-dependent defense responses. Moreover, defense-related transcription factors and genes involved in biosynthetic pathway of green leaf volatiles and volatile terpenes were also activated, whereas growth-related photosynthesis and SA-dependent defenses were suppressed. In addition, a terpene synthase gene *PbeOCS* was present in the RNA-seq data and functionally characterized *in vitro*. Recombinant PbeOCS catalyzed GPP as substrate to produce (*E*)-β-ocimene and the compound had significant impacts on choice behavior and growth of *S. litura* larvae in insect bioassays. These findings should contribute to generating valuable markers for further breeding insect-resistant pear varieties and developing an environmentally friendly strategy for pest control.

## Data availability statement

The original contributions presented in the study are publicly available. This data can be found here: NCBI, PRJNA783361 and GenBank, OK635579.

## Author contributions

WS and YZ conceived and designed the study. XZH, HZ, HL, MW, XG, and EL performed the experiments and analyzed the data. XQH, CZ, and LL contributed the resources. XZH, HZ, WS, and YZ wrote the manuscript. All authors read and approved the submitted manuscript.

## Funding

This work was supported by the National Natural Science Foundation of China (32272638, 31972338, 31772176, 31701800), and the Open Fund Project of State Key Laboratory for Biology of Plant Diseases and Insect Pests (SKLOF201901).

## Conflict of interest

The authors declare that the research was conducted in the absence of any commercial or financial relationships that could be construed as a potential conflict of interest.

## Publisher’s note

All claims expressed in this article are solely those of the authors and do not necessarily represent those of their affiliated organizations, or those of the publisher, the editors and the reviewers. Any product that may be evaluated in this article, or claim that may be made by its manufacturer, is not guaranteed or endorsed by the publisher.

## Supplementary material

Supplementary Figure 1 Principal component analysis of transcriptomic data.

Supplementary Figure 2 Number of up- and downregulated genes in *Pyrus betuleafolia* leaves after *Spodoptera litura* infestation.
