# Supplementary material for: Functional characterization of a terpene synthase responsible for (E)-β-ocimene biosynthesis identified in Pyrus betuleafolia transcriptome after herbivory
Source: Front Plant Sci. 2022 Nov 21;13:1077229. doi: 10.3389/fpls.2022.1077229 (PMC9720175; doi:10.3389/fpls.2022.1077229)
Supplement: Supplementary file 1 [file Table_1.docx]

**Tables S1.** **Primers used to isolate *PbeOCS* and in qRT-PCR analysis in this study.**

| **Name** | **Sequence (5’→3’)** | **Usage (5’→3’)** |
| --- | --- | --- |
| PbeOCS-FP  PbeOCS-RP  Tubulin-QFP  Tubulin-QRP  PbeOCS-QFP  PbeOCS-QRP  GWHGAAYT034025-QFP  GWHGAAYT034025-QRP  GWHGAAYT042495-QFP  GWHGAAYT042495-QRP  GWHGAAYT053166-QFP  GWHGAAYT053166-QRP  GWHGAAYT045618-QFP  GWHGAAYT045618-QRP  GWHGAAYT000808-QFP  GWHGAAYT000808-QRP  GWHGAAYT011340-QFP  GWHGAAYT011340-QRP  GWHGAAYT005252-QFP  GWHGAAYT005252-QRP | ATGCCTAATAATACGCCTCTTC  TTAAATGGGATCGACAATCACT  TGGGCTTTGCTCCTCTTAC  CCTTCGTGCTCATCTTACC  CGTGGCAGTCTAAATGTTTC  CGATTCTTTGCTCTTGTGTC  GTTGGAGCTGTTCAGTTAGA  TTAGCTTCTTCAACCCACTC  ATGACACTAGGTTTGCATGT  GTTGGTAAAGCTGAAGAGGA  AACAATGTTGATCACTCCGA  GATGATCAAAACCCCAGTCT  GCATAGCCGAGATCATAGAG  TCATTGTCTGACAGCATTGA  ACATTCTTGGCATCAGGATT  GCATGTCTCATCCTTGGTAT  TTTACTGATCACTTGACGCA  TTCAATTTGAGGTCCTAGCC  GTTTTCCCAGAGCTCTTGT  GGCAATTCTGTATCCCTCAA | isolation  isolation  qRT-PCR  qRT-PCR  qRT-PCR  qRT-PCR  qRT-PCR  qRT-PCR  qRT-PCR  qRT-PCR  qRT-PCR  qRT-PCR  qRT-PCR  qRT-PCR  qRT-PCR  qRT-PCR  qRT-PCR  qRT-PCR  qRT-PCR  qRT-PCR |
