# Supplementary figures and images for: Functional characterization of a terpene synthase responsible for (E)-β-ocimene biosynthesis identified in Pyrus betuleafolia transcriptome after herbivory
Source: Front Plant Sci. 2022 Nov 21;13:1077229. doi: 10.3389/fpls.2022.1077229 (PMC9720175; doi:10.3389/fpls.2022.1077229)

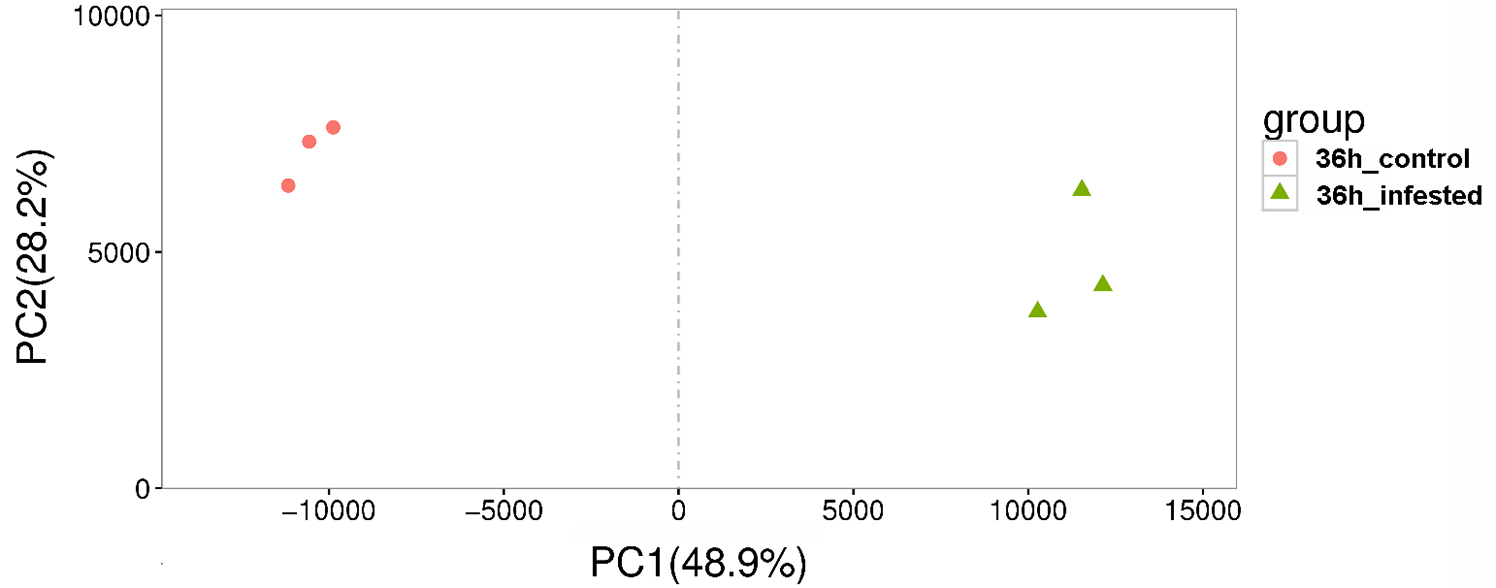

Supplement: Supplementary Figure 1 — Principal component analysis of transcriptomic data. [file Image_1.jpeg]

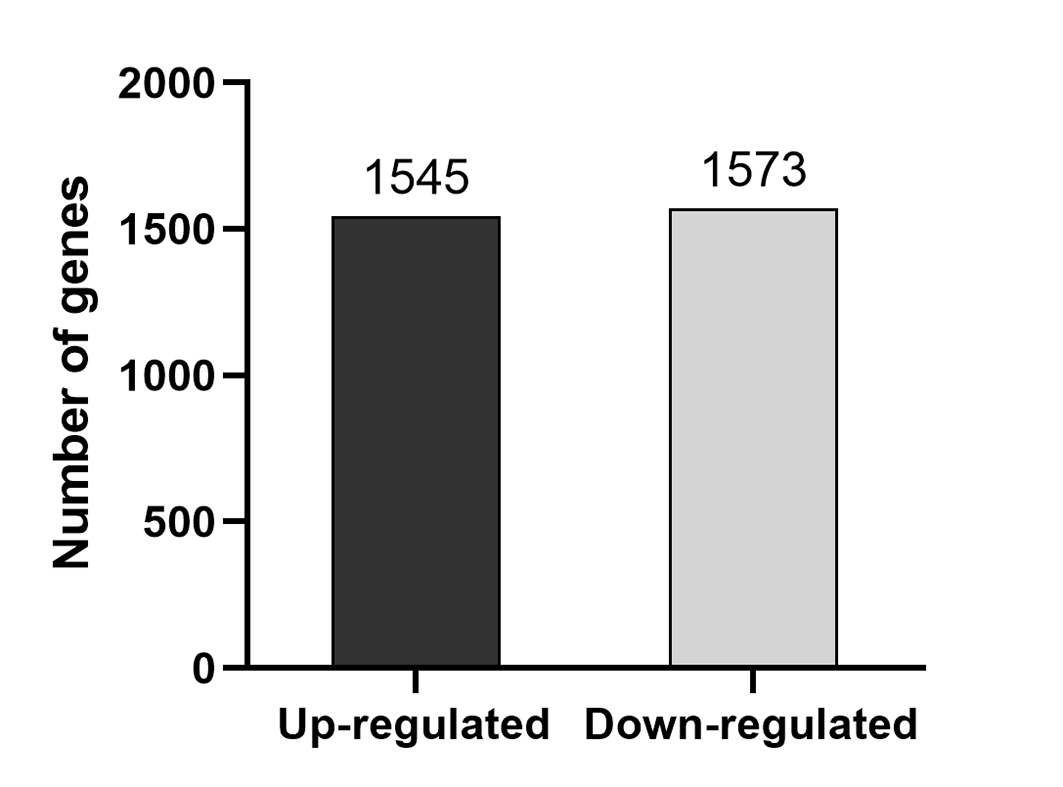

Supplement: Supplementary Figure 2 — Number of up- and downregulated genes in Pyrus betuleafolia leaves after Spodoptera litura infestation. [file Image_2.jpeg]
